# Poland-Mobius syndrome in an infant girl

**DOI:** 10.4103/0256-4947.57174

**Published:** 2009

**Authors:** Khalid A. Al-Mazrou, Yazeed A. Al-Ghonaim, Abdulrhman I. Al-Fayez

**Affiliations:** a From the Otolaryngology Department, King Saud University, Riyadh, Saudi Arabia; b From the Otolaryngology Department, King Abdulaziz Medical City, Riyadh, Saudi Arabia

## Abstract

Mobius syndrome is a rare condition of unclear origin, characterized by a unilateral or bilateral congenital facial weakness with impairment of ocular abduction, which is frequently associated with limb anomalies. Poland described a condition in which there was unilateral absence of pectoralis major muscle and ipsilateral syndactyly. The combination of Poland-Mobius syndrome is rare, with an estimated prevalence 1:500 000. We describe a case of Poland-Mobius syndrome in association with congenital bilateral vocal fold immobility. To our knowldge, this is the first report of such an association between Poland-Mobius syndrome and congenital bilateral vocal fold immobility.

Poland-Mobius syndrome is a rare congenital disorder that includes combination features of Poland and Mobius syndromes. Poland syndrome consists of absence of pectoralis major muscle, syndactyly, barchydactyly and hypoplasia of the hands.^1^ Mobius syndrome includes a variable degree of facial paralysis with an inability to abduct the eyes beyond midpoint.^1^ Unilateral or bilateral vocal fold paralysis is a significant cause of stridor in infant and children. Neonatal bilateral vocal fold paralysis may be caused by birth trauma, asphyxia, or central nervous system (CNS) anomalies.^2^ Other recognizable syndromes such as Down syndrome, 22q deletion syndrome, and Robinow syndrome can present with bilateral vocal fold paralysis.^3^ In most cases of Mobius syndrome, unilateral vocal fold immobility is a frequent feature. We report an infant with macrocephaly, hypertelorism, low set ears, a restricted mouth opening, an expressionless face, upper limb anomalies and bilateral talipas equino varus. This may be the first reported case of Poland- Mobius syndrome associated with bilateral vocal fold immobility.

## CASE

**Figure 1:**
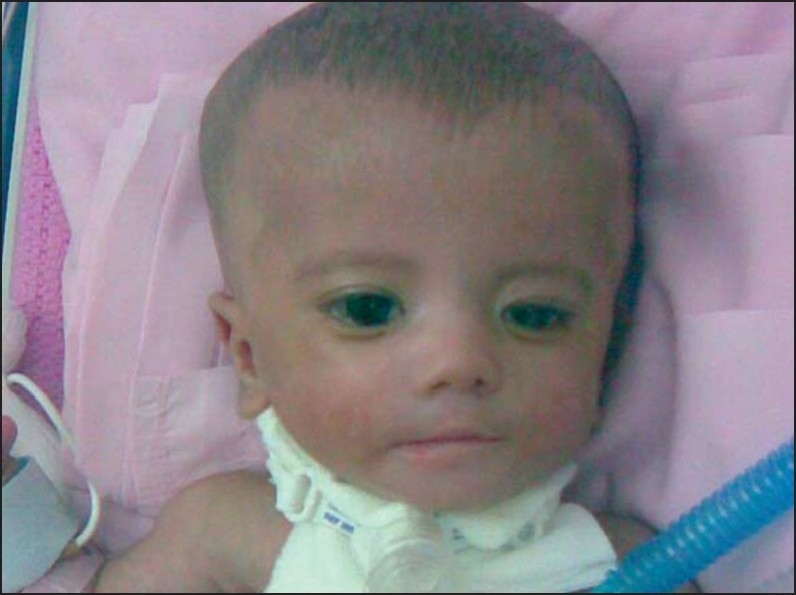
A photo of the infant showing some of the dysmorphic features (hypertelorism, low set ears, a restricted mouth opening and an expressionless face).

**Figure 2:**
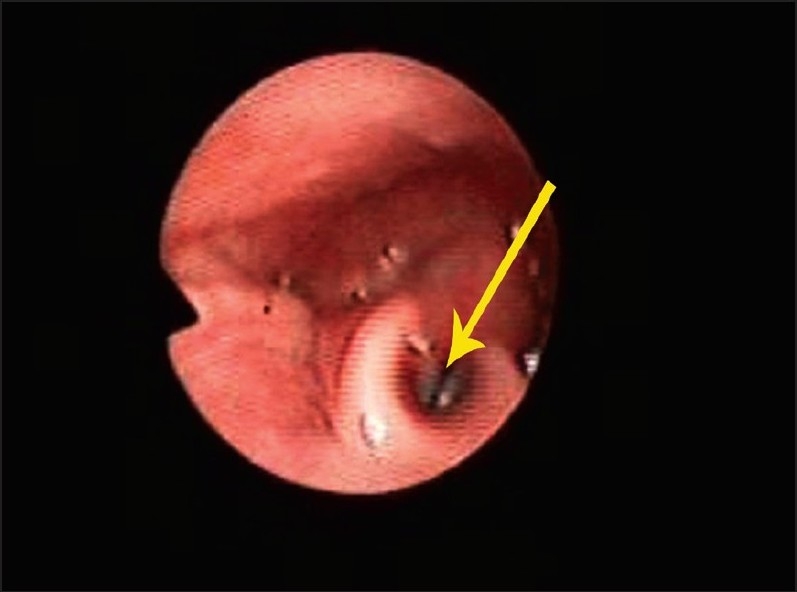
A still image of flexible nasoendoscopy showing the immobile vocal folds (arrow).

A 20-day-old Saudi infant girl referred for evaluation and management of respiratory distress to the Pediatric Otolaryngology service at King Abdulaziz Medical City. The patient was the first of identical (monozygous) female twins born at 34 weeks of gestation by caesarean delivery due to breech presentation. The Apgar score was 2 and 8 at 1 and 5 minutes, respectively. The weight of the girl was 1.85 kg, height 42.5 cm and her head circumference was 34 cm. The mother was G2P0 and her pregnancy resulted from *in vitro* fertilization (IVF). A positive history of consanguinity (first degree relatives) was reported. There were no dysmorphic features in the parents or siblings. The second twin had been discharged on the first day in good health status with no congenital abnormalities. At birth, the baby had cyanosis and respiratory distress. She was immediately intubated with difficulty and transferred to the neonatal intensive care unit (NICU). Examination showed a dysmorphic baby with macrocephaly (box-like skull) and a prominent parietal region with a high forehead. The baby also had hypertelorism, low set ears, a restricted mouth opening and an expressionless face (Figure 1). Her right upper limb appeared to be hypoplastic with 2^nd^, 3^rd^ and 4^th^ partial syndactyly and barchydactyly. Her right pectoralis major muscle was hypoplastic. The left hand had features of camptodactyly. Her lower limbs revealed bilateral talipes equinovarus. A possible diagnosis of Poland-Mobius syndrome was suggested. She was extubated after 3 days and transferred to the intermediate care unit of neonates (ICN). One week later, she developed severe respiratory distress and desaturation to 80%. Flexible nasoendoscopy showed moderate pharyngeomalacia and bilateral vocal fold immobility (Figure 2). Feeding was through the orogastric tube. Magnetic resonance imaging (MRI) of the brain showed normal brain tissue. Swallowing could not be evaluated due to fixed jaws.

At the age of one month, a tracheotomy was needed because of recurrent attacks of respiratory distress and the anticipated possibility of difficult intubation. At the age of 2 months, the child underwent fundoplecation and gastrostomy tube because of dysphagia and gastro esophageal reflux disease (GERD). Chromosomal analysis of both twins showed a normal 46XX karyotype.

## DISCUSSION

Mobius syndrome was first reported in 1880 by Von Greave and in 1888 by Paul Julius Mobius.^4^ The syndrome consists of congenital complete or partial facial nerve paralysis with or without paralysis of other cranial nerves. The most common cranial nerve involved is the 6^th^ cranial nerve (abducent). The oculomotor and trochlear can also be involved. It is also known as congenital facial diplegia, nuclear agenesis, congenital nuclear hypoplasia, congenital occulofacial paralysis and congenital abducens—facial paralysis.^1^ According to the pathologic changes, Mobius syndrome is classified into four groups.^5^ Group I is characterized by simple hypoplasia or atrophy of cranial nerve nuclei, presumably as a result of embryonic maldevelopment. Group II results from primary lesions in the peripheral portion of the cranial nerves. Group III is due to focal necrosis in the brain stem nuclei. Group IV consists of patients without lesions in the central nervous system or cranial nerves but showed features of primary myopathy.

The Mobius syndrome represents a distinctive malformation syndrome, but its etiology remains undetermined. Numerous theories exist concerning the primary underlying pathogenesis. Intrauterine vascular etiology involves disruption of the flow in the basilar artery or premature regression of the primitive trigeminal artries.^6^ Toxic or a degenerative material which involves the nuclei of the affected nerves was suggested as alternative theory. Congenital inherited hypoplasia or agenesis of the CN nuclei, and infectious factors have been also proposed as important factors.

Poland syndrome was described in 1841 by Alfred Poland as unilateral absence of pectoralis major muscle and ipsilateral dermal syndactyly of the hand.^7^ Other anomalies associated with this syndrome include hypoplasia of the forearm, hypoplasia of the breast, agenesis of the nipple, rib cage deformities, bilateral epicanthus and talipes equinovarus.^1^ Boys are affected more than girls. The reported frequency is 1:20 000.^1^ Ten percent of all cases of hand syndactyly may have the Poland sequence. The syndactyly is usually in the right hand as in our patient. Poland syndrome is not an inheritable condition nor one that arises from events during pregnancy, which was established in a detailed study of twins affected with Poland syndrome using DNA evidence to establish that they were identical.^8^ Furthermore, since the both of the embryos, fetuses and newborns have been exposed to identical physical and chemical conditions, there were no environmental teratogenic sources implicated. This non-genetic and non-teratogenic etiology suggests that Poland syndrome is entirely sporadic.

The combinations of Poland and Mobius anomalies are rarely described in the literatures with an estimated prevalence of 1:500 000.^1^,^9^–^11^ Some authors believe this association is an independent syndrome. Others think that Poland, Mobius and Poland-Mobius syndromes are variations of the same condition.^9^

Unilateral or bilateral vocal fold paralysis is a significant cause of stridor in infants and children.^2^ Bilateral vocal fold paralysis is the second most common cause of stridor in the neonate. Flexible laryngoscopy is the main method for the diagnosis of vocal fold immobility. Neonates and infants with suspected vocal fold paralysis must be carefully evaluated for underlying diseases. Neonatal bilateral vocal fold paralysis may be caused by birth trauma, asphyxia or central nervous system anomalies.^2^ It has been also described in some recognizable syndromes, including Down syndrome, 22q deletion syndrome, Robinow's syndrome and cerebro-oculo-facio-skeletal syndrome.^3^ Up to our knowledge, no previous cases of Poland-Mobius syndrome with congenital bilateral vocal fold paralysis have been reported in the English literature.
